# Health Status and Patient Satisfaction after Corneal Graft: Results from the Corneal Transplant Epidemiological Study

**DOI:** 10.1155/2012/230641

**Published:** 2012-04-24

**Authors:** Adriano Fasolo, Cristina Capuzzo, Michela Fornea, Anna Chiara Frigo, Cristina Monterosso, Alfonso Zampini, Antonio Avarello, Alessandro Galan, Sandro Sbordone, Adele Elisabetta Ragucci, Claudio Gorla, Francesco Grigoletto, Diego Ponzin

**Affiliations:** ^1^Fondazione Banca degli Occhi del Veneto, Via Paccagnella 11, 30174 Zelarino, Venezia, Italy; ^2^Dipartimento di Scienze Cardiologiche, Toraciche e Vascolari, Università degli Studi di Padova, 35131 Padova, Italy; ^3^U.O. Oculistica, Ospedale dell'Angelo, 30174 Zelarino, Venezia, Italy; ^4^U.O. Oculistica, Ospedale S. Antonio, 35127 Padova, Italy; ^5^Clinica Oculistica, Policlinico Federico II, 80131 Napoli, Italy; ^6^U.O. Oculistica, Ospedale Ca' Foncello, 31100 Treviso, Italy

## Abstract

*Purpose*. To evaluate effects of corneal transplantation on the health-related quality of life and patients' satisfaction. *Methods*. Patients scheduled for elective penetrating or anterior lamellar keratoplasty completed by telephone interview the SF-12 Health Survey, before and one year after surgery, and a 6-item questionnaire on the satisfaction for graft outcomes. *Results*. The two questionnaires were answered by 1,223 patients. Transplantation did not influence the PCS-12 in males (ES = −0.01) and had a negative effect in females (ES = −0.18). Both sexes improved their MCS-12 (ES = 0.18 and 0.23, resp.). The majority of patients (83.1%) were satisfied by the outcome of the graft. *Conclusions*. This is the first report on the use of the SF-12 and one of the few that assess quality of life in patients after corneal transplantation. We showed that grafting improves patients' health-related quality of life results of patients, influencing mental health (i.e., psychological attitude, social interaction, and emotions) with minor effects on physical health (limitation, pain, and vitality).

## 1. Introduction

Health-related quality of life (HRQOL) refers to issues associated with a person's perception of physical and mental well-being, which can be affected by illness and medical treatment [1]. The use of standardized instruments increases the capability to convert patients' subjective opinions, ratings, and reports concerning their health, in an informative way, which can be used to assess the overall health status and to monitor changes in HRQOL [2]. Moreover, the assessment of patient's HRQOL plays an increasingly important role in the evaluation of primary or secondary outcomes of treatments, as documented in a number of clinical studies [3].

Measuring quality of life in ophthalmology has been confirmed as an important tool for evaluating eye diseases and the impact of interventions [4–6].

Some corneal diseases, usually in the late stage, require corneal transplantation [7], which is mostly performed on an elective basis. Criteria for success primarily depend on the indication for surgery, that is, improvement of visual acuity, pain relief, and maintenance of the structural integrity of the eye [8]. The clinical outcome is normally graded by the caregiver and might not completely reveal the overall benefits, or detriments, perceived by the patient [9].

Actually, patients can easily appraise the graft and estimate the overall results on daily living and in respect of their expectations. Furthermore, while corneal transplantation is performed to treat a corneal disease, surgeons have a reasonable expectation that surgery will lead to a significant improvement in their patient's overall physical and mental health [10].

However, the global impact of the corneal transplantation on the patient's health status or satisfaction is largely unknown.

Studies that have considered the issue of health status in corneal transplantation have used vision-targeted health-related quality of life instruments (VF-14, VFQ) [11, 12] or generic health status questionnaires (e.g., SF-36 Health Survey) [13], within longitudinal [14–16] or cross-sectional studies [17, 18].

Despite the disparities in the number of patients, such studies have shown that postgraft visual function significantly affects health status and that visual acuity in the nonoperated eye is the strongest factor associated with improvement of visual function.

Only two published surveys [19, 20] deal with patients' satisfaction after corneal transplantation, and they point out that patients' education and pregraft expectations affect the perception of success more than the actual clinical outcome.

In the framework of the Corneal Transplant Epidemiological Study (CORTES) [21, 22], that was performed to understand corneal transplantation in Italy, and to examine the long-term graft survival, we assessed the effect of such surgery on patients' HRQOL and satisfaction. Moreover, the HRQOL results were compared with those of a representative sample of healthy subjects of the Italian population.

## 2. Materials and Methods

### 2.1. Patients

The corneas distributed by the Veneto Eye Bank Foundation (about 2,000 per year in the 2002–2008 period) represent approximately 40% of corneal tissues transplanted in Italy [23].

All the consecutive patients scheduled for elective penetrating or anterior lamellar keratoplasty during the CORTES recruitment period (October 2001–October 2004) were considered eligible for HRQOL and satisfaction evaluation.

Exclusion criteria were age below 18 years, presence of invalidating diseases that could cause difficulties during the interview, and patient or surgeon refusal of the interview.

HRQOL data were collected through standardized telephone interviews, carried out by personnel of the CORTES Follow-Up Unit during the week prior to corneal transplantation and one year following surgery. Assessment of patient satisfaction was completed at the time of the second interview.

Patients' clinical history, type of intervention, postoperative clinical assessment, adverse reactions and complications, and visual acuity and graft condition after one year were obtained from the CORTES database.

The study protocol was approved by the Ethical Committee of the University of Padua. Full details about the study and the list of contributing surgeons have been reported elsewhere [21].

### 2.2. The SF-12 Health Survey

The SF-12 Health Survey [24] is a multipurpose, generic 12-item questionnaire developed from the Short Form-36 (SF-36) that is one of the most widely used health status evaluation tools. The SF-12 provides a shorter but still valid and reliable alternative to the SF-36 for use in large samples.

The questionnaire, validated in Italian [3, 25] and used previously in ophthalmic research [26], estimates scores for eight health concepts: physical functioning, role limitations due to physical health problems, role limitations due to emotional health problems, mental health (scored using two items each) bodily pain, general health, vitality, and social functioning (estimated using one item each). SF-12 produces two synthetic measures evaluating physical (limitation, pain, and vitality) and mental (psychological attitude, social interaction, and emotions) aspects of health, the Physical Component Summary (PCS-12) and the Mental Component Summary (MCS-12). These measures correlate with the SF-36 in the 0.94–0.97 range [25].

Validation studies showed that PCS-12 and MCS-12 range from 11 to 70 and from 7 to 72, respectively. Both summary scores are standardized to have a mean of 50 and a standard deviation of 10, with higher scores indicating better health status perception [27].

SF-12 summary scores were examined in relation to sociodemographic and graft-related variables. On the basis of previous results [27] it was expected that females, elderly people, widows, divorced people, and those who have a lower education or are unemployed would report poorer health status.

### 2.3. Patient Satisfaction Assessment

To evaluate satisfaction we used 6 items on expectation and satisfaction comprised in the 24-item questionnaire designed by Williams et al. at Flinders University of South Australia, Adelaide [19]. Satisfaction questionnaires were administered to patients one year after surgery following the SF-12 interview.

### 2.4. Statistical Analysis

Patients who completed the two SF-12 interviews and the satisfaction questionnaire were considered for evaluation.

Analyses on the SF-12 were conducted with SAS version 9.1.3 statistical software (SAS Institute Inc., Cary, NC, USA) following the methods reported in the user manual [27].

Mean values and 95% confidence intervals for PCS-12 and MCS-12 were calculated according to patients' social and disease-related characteristics reported to influence the HRQOL.

Results of descriptive analyses are expressed as mean and standard deviation (SD) for quantitative variables and as counts and percentages for categorical variables, unless otherwise specified. Chi square test was used to compare categorical variables in eligible and interviewed patients and Wilcoxon-Mann-Whitney nonparametric test to compare median of ages. Chi square test was also used to evaluate differences among patients in relation to their satisfaction after transplantation. All *P* values are two tailed.

In order to assess the magnitude and meaning of health status changes we use the effect size (ES) calculated by taking the differences between means before treatment and after treatment and dividing it by the SD of the same measure before treatment. As suggested by Kazis et al. [28], small magnitude of change is indicated by an effect size of 0.2, moderate by 0.5 and large by 0.8. 

## 3. Results and Discussion

### 3.1. Patients' Demography, Baseline, and Assessments One Year following Surgery

A total of 2,329 patients (44.7%) were considered to be eligible over the total of 5,210 that underwent corneal transplantation during the study period.

Among the eligible patients, 785 (33.7%) missed the baseline interview (529 did not answer the phone call, 92 declined the consent, 89 underwent surgery at an earlier date, 56 had linguistic problems, and 19 did not answer for other reasons). A further 321 patients (13.8%) missed the one-year-after-surgery interview and were therefore excluded from the analysis (185 did not answer the phone call, 92 refused the interview; 31 had been already regrafted because of graft failure, and 13 had difficulty in carrying on the interview).

Ultimately, 1,223 patients (52.5%) referring to 96 ophthalmic surgeons in 66 centers answered the two SF-12 and the satisfaction questionnaire.

Sociodemographic and clinical characteristics of the entire cohort, eligible and interviewed patients are reported in Table 1.

A significant difference between interviewed and eligible patients (*P* = 0.026) was found only for the variable “reasons for graft.”

Best corrected visual acuity (BCVA) before transplantation is reported in Table 4. One year after surgery, 399 (32.6%) patients had BVCA of 8/10 or greater, 667 (54.6%) were between 3/10 and 7/10, and 91 (12.8%) had a visual acuity less than 2/10.

A total of 54 (4.4%) grafts failed and one or more adverse reactions/complications (AE/C), for example, IOP elevation, suture-related problems, and rejection episodes, occurred in 188 (15.4%) patients within the first year of follow-up. 

### 3.2. Overall Health Status Graded by Patients

The answers to the first item of SF-12 at baseline (*in general, would you say your health is excellent, very good, good, fair, poor*) showed that the large majority of males and females grade their health in general as *excellent/very good* or *good* (Tables 2 and 3).

One year after surgery, males considered their health status to be improved (20.2%), worse (16.6%), or unchanged (63.2%), compared to baseline. In the females group, the corresponding figures were 18.3%, 20.4%, and 61.3%.

### 3.3. Physical (PCS-12) and Mental (MCS-12) Health Status at Baseline and after One Year

Scores given by males were higher than scores given by females, both at baseline and after one year.

Meaningful differences were observed within both SF-12 summary component scores across the distribution of demographic and clinical variables (Table 4).

Since the reliability of SF-12 summary scores is not assured for small numbers, results in categories with less than 100 cases should be considered with caution (i.e., some age groups, widow/divorced, no education, student, jobless, and visual acuity higher than 5/10).

For the patient sample as a whole, the mean PCS-12 remained unchanged (ES = 0.09) but variations of MCS-12 suggest improvement of HRQOL with a small magnitude of change (ES = 0.19). Considering males and females separately, transplantation did not influence physical health in males (PCS-12 ES = −0.01) and had a negative effect in females (PCS-12 ES = −0.18). However, mental health improved in both genders after one year (MCS-12 ES = 0.18 and 0.23, resp.).

As far as patients' age is concerned, PCS-12 showed slight changes while MCS-12 increased after transplantation in several age intervals of both genders.

Physical health was not affected by almost all demographic and socioeconomic variables or decreased only in some cases (married people, people with low level of education, and retired people) with negative ES values ranging from 0.16 to 0.23. On the other hand, mental health substantially increased, with positive MCS-12 ES values from 0.16 to 0.28.

Systemic diseases and AE/C reported in follow-up were associated with a poorer PCS-12 after corneal transplantation (ES = −0.21 and −0.24, resp.) but all health and eye-related variables correlated positively with increased MCS-12 values at one year (ES ranging from 0.10 to 0.23).

### 3.4. Patient Satisfaction and SF-12 Comparison with the Italian Validation Survey

The majority of patients were satisfied with the graft and would decide to undergo surgery again. They considered their graft had been worthwhile (Table 5). However, 24.0% of patients believed that the outcome did not match their expectations, and 22.2% reported that they had experienced more complications than expected.

No differences were found in the satisfaction with graft outcome between genders (*P* = 0.5), while AR/C in follow-up and a visual acuity of ≤2/10 after one year were found to be significantly associated with unhappiness with graft outcome (*P* < 0.001).

Distributions of answers to the sixth item of the questionnaire (*Overall, how much satisfied are you with your graft?*) showed 10.3% of patients to be unsatisfied and 2.9% to be uncertain (Table 6). No significant differences between sexes were found (*P* = 0.3).

As assessed in the SF-12 validation study on more than 61,000 subjects [3, 27], females show poorer HRQOL than males with mean (SD) values of PCS-12 and MCS-12 of 49.1 (10.1) and 48.9 (9.4) and 51.1 (8.7) and 51.5 (9.1), respectively.

In the same study, about 30,000 healthy subjects reported PCS-12 values of 53.7 (6.0) and MCS-12 of 52.8 (7.9). As expected, diseased patients showed clear decreases of both summary components, with PCS-12 scores ranging from 45.9 (11.0) to 37.2 (11.1) in patients with migraine and past stoke and MCS-12 scores ranging from 46.0 (11.5) to 33.8 (10.8) in patients with prostatic hypertrophy and central nervous system pathologies, respectively. In the present study, patients waiting for corneal transplantation showed PCS-12 of 50.8 (6.9) and MCS-12 of 48.4 (10.5) with SF-12 values lower than those of healthy population in both genders (Table 4).

## 4. Conclusions

This is the first report on the use of the SF-12 Health Survey in corneal transplantation and one of the few longitudinal studies that have assessed changes in the HRQOL in patients who have undergone corneal grafting. The study showed that the graft improves patients' HRQOL and influences mental health more than physical health.

Consistently with almost all the surveys using the HRQOL, females showed poorer quality of life scores than males. Moreover, our study showed that physical health in females was negatively affected by corneal transplantation while grafts in males did not change the PCS-12 baseline values.

Since no difference in graft outcomes is expected between the genders and satisfaction with grafts was the same for males and females, the inconsistency of PCS-12 results could highlight differences in perceptions between the sexes with respect to the impact of grafting on physical health. Actually, there are no obvious reasons why young or middle-aged patients—that generally maintain a normal life style, with minor limitation, while waiting for surgery—should have reduced their physical health after the graft.

Conversely, the significant improvement of mental health in both sexes shows that although the graft primarily aims to improve visual function, corneal transplantation largely affects psychological and emotional aspects of self-perceived health.

This result parallels the recent finding by Kymes and coworkers [5] who showed that keratoconus significantly impairs mental health more than all other scales of the Visual Function Questionnaire.

The restoration of sight is the most important purpose of corneal grafting. However, since successful corneal transplantation can improve both health and lifestyle of patients, their personal feelings should be considered as a central issue in their overall assessment.

## Figures and Tables

**Table 1:** Sociodemographic and clinical characteristics of patients.

	Cohort No. (%)	Eligible No. (%)	Interviewed No. (%)*	*P *value^†^
	5,210 (100)	2,329 (44.7)	1,223 (52.5)	
Gender				0.858
Male	3,040 (58.3)	1,362 (58.5)	719 (58.8)	
Female	2,170 (41.7)	967 (41.5)	504 (41.2)	
Age (y)				
Mean (SD)	49.0 (19.8)	46.8 (17.9)	45.8 (16.9)	
Male	46.0 (19.2)	44.1 (17.6)	43.9 (16.8)	
Female	53.1 (19.9)	50.4 (17.8)	48.6 (16.6)	
Median	46.0	44.0	43.0	0.266
Male	42.0	40.0	41.0	0.972
Female	53.0	49.0	47.0	0.084
Marital status				
Married	n.c.	n.c.	676 (57.1)	
Single			412 (34.8)	
Widow			62 (5.2)	
Divorced			34 (2.9)	
*Unreported *			*39*	
Education				
High school	n.c.	n.c.	457 (39.4)	
Secondary school			311 (26.8)	
Primary school			227 (19.6)	
Degree			143 (12.3)	
None			22 (1.9)	
*Unreported *			*63*	
Working status				
Partial or full time	n.c.	n.c.	632 (55.6)	
Retired			222 (19.5)	
House keeper			181 (15.9)	
Student			65 (5.7)	
Jobless			36 (3.2)	
*Unreported *			*87*	
Reason for graft				0.026
Optical	4,183 (90.8)	1,994 (92.5)	1,085 (94.7)	
Tectonic	228 (4.9)	84 (3.9)	26 (2.3)	
Pain reduction	197 (4.3)	78 (3.6)	34 (3.0)	
*Unreported *	*602*	*173*	*78*	
Type of keratoplasty				0.227
Penetrating	4,696 (90.1)	2,157 (92.6)	1,146 (93.7)	
Anterior lamellar	514 (9.9)	172 (7.4)	77 (6.3)	
Clinical indication				0.386
Ectasia/thinning	2,550 (48.9)	1,298 (55.7)	730 (59.7)	
Regraft (*related or not to rejection*)	674 (12.9)	304 (13.0)	148 (12.1)	
Pseudophakic corneal edema	594 (11.4)	195 (8.4)	87 (7.1)	
Primary endotheliopathies	335 (6.4)	140 (6.0)	82 (6.7)	
Mechanical trauma	174 (3.3)	76 (3.3)	36 (3.0)	
Viral/postviral keratitis	129 (2.5)	50 (2.2)	25 (2.0)	
Stromal corneal dystrophy	79 (1.5)	35 (1.5)	23 (1.9)	
Aphakic corneal edema	102 (2.0)	30 (1.3)	14 (1.2)	
Optical/refractive	67 (1.3)	36 (1.6)	14 (1.2)	
Microbial/postmicrobial keratitis	76 (1.5)	32 (1.4)	13 (1.1)	
Noninfectious ulcerative keratitis	177 (3.4)	38 (1.6)	10 (0.7)	
Chemical injures	36 (0.7)	8 (0.3)	3 (0.2)	
Other (*leucoma by unknown cause; silicone keratopathy; inborn opacities*)	217 (4.2)	87 (3.7)	38 (3.1)	
Previous keratoplasty				0.369
No	4,094 (86.1)	1,896 (86.2)	1,020 (87.3)	
Yes	663 (13.9)	303 (13.8)	148 (12.7)	
*Unreported *	*453*	*130*	*55*	
Previous surgery (*except keratoplasty*)				0.120
No	3,251 (68.3)	1,610 (73.2)	884 (75.7)	
Yes	284 (31.7)	589 (26.8)	284 (24.3)	
*Unreported *	*453*	*130*	*55*	
Other ocular diseases				0.345
No	3,700 (77.2)	1,734 (77.0)	939 (78.5)	
Yes	1096 (22.8)	515 (23.0)	257 (21.5)	
* Unreported*	*414*	*81*	*27*	
Systemic diseases				0.455
No	3,657 (78.6)	1,723 (77.8)	933 (78.9)	
Yes	997 (21.4)	491 (22.2)	249 (21.1)	
* Unreported*	*556*	*116*	*41*	

*Proportion of the eligible patients; n.c.: data not collected; ^†^comparison between interviewed and eligible.

**Table 2:** Health in general at baseline (*T*_0_) and after one year (*T*_1_) in males.

		*T*_1_			
		Excellent/very good *N* (%)	Good *N* (%)	Fair/poor *N* (%)	Total *N* (%)
*T*_0_	Excellent/very good	142 (19.7)	81 (11.3)	7 (1.0)	230 (32.0)
	Good	111 (15.4)	293 (40.8)	31 (4.3)	435 (60.5)
	Fair/poor	6 (0.8)	29 (4.0)	19 (2.7)	54 (7.5)

	Total	259 (35.9)	403 (56.1)	57 (8.0)	719 (100.0)

**Table 3:** Health in general at baseline (*T*_0_) and after one year (*T*_1_) in females.

		*T*_1_			
		Excellent/very good *N* (%)	Good *N* (%)	Fair/poor *N* (%)	Total *N* (%)
*T*_0_	Excellent/very good	46 (9.1)	54 (10.7)	1 (0.2)	101 (20.0)
	Good	39 (7.8)	211 (41.9)	48 (9.5)	298 (59.2)
	Fair/poor	6 (1.2)	47 (9.3)	52 (10.3)	105 (20.8)

	Total	91 (18.1)	312 (61.9)	101 (20.0)	504 (100.0)

**Table 4:** PCS-12 and MCS-12 (*mean* and 95% CI) and effect size (ES) by subgroups.

		PCS-12			MCS-12		
	*N*	*T*_0_	*T*_1_	ES*	*T*_0_	*T*_1_	ES*
Total	1,223	50.8 (50.4–51.2)	50.2 (49.8–50.6)	0.09	48.4 (47.8–49.0)	50.4 (49.8–51.0)	0.19

Gender							
Male	719	51.5 (51.0–51.9)	51.4 (50.9–51.9)	−0.01	50.8 (50.1–51.4)	52.4 (51.7–53.1)	0.18
Female	504	49.9 (49.3–50.6)	48.5 (47.8–49.2)	−0.18	45.0 (44.0–46.0)	47.6 (46.6–48.6)	0.23
Male, age groups (y)							
18–24	88	52.8 (51.5–54.2)	52.5 (51.3–53.7)	−0.06	49.8 (47.8–51.9)	51.0 (48.9–53.0)	0.12
25–34	178	51.9 (51.0–52.8)	52.7 (51.9–53.5)	0.13	50.5 (49.3–51.7)	53.1 (52.0–54.2)	0.33
35–44	155	51.5 (50.6–52.5)	51.5 (50.5–52.6)	—	49.8 (48.2–51.3)	51.2 (49.7–52.6)	0.15
45–54	111	52.1 (50.8–53.3)	51.3 (50.0–52.6)	−0.11	49.3 (47.3–51.2)	51.3 (49.4–53.1)	0.19
55–64	85	50.3 (49.1–51.6)	51.0 (49.7–52.1)	0.10	52.2 (50.1–54.2)	54.9 (53.3–56.6)	0.29
65–74	59	50.8 (49.6–52.1)	48.4 (46.7–50.0)	−0.49	55.0 (53.2–56.8)	54.6 (52.2–57.0)	−0.06
≥75	43	48.4 (46.1–50.7)	49.4 (47.0–51.8)	0.13	52.5 (49.4–55.7)	51.9 (48.1–55.6)	−0.06
Female, age groups (y)							
18–24	27	52.6 (50.1–55.1)	52.2 (49.9–54.5)	−0.07	43.6 (39.1–48.2)	50.4 (46.1–54.6)	0.60
25–34	108	50.3 (48.8–51.8)	50.8(49.6–52.0)	0.06	42.1 (40.0–44.2)	45.7 (43.6–47.8)	0.33
35–44	91	51.2 (49.6–52.7)	49.9 (48.3–51.5)	−0.17	44.9 (42.6–47.1)	48.6 (46.4–50.9)	0.35
45–54	101	50.5 (48.9–52.0)	49.4 (48.0–50.8)	−0.14	45.1 (42.9–47.3)	48.0 (45.8–50.2)	0.26
55–64	72	49.8 (48.3–51.3)	47.4 (45.3–49.4)	−0.38	47.6 (44.8–50.3)	48.2 (45.6–50.8)	0.05
65–74	70	48.4 (46.6–50.1)	45.7 (43.6–47.9)	−0.36	48.0 (45.7–50.3)	49.4 (46.2–52.5)	0.14
≥75	35	45.7 (43.1–48.3)	41.6 (38.2–44.9)	−0.52	43.8 (39.9–47.7)	43.3 (39.5–47.0)	−0.05
Marital status							
Married	676	51.1 (50.6–51.5)	50.0 (49.4–50.5)	−0.16	48.8 (48.1–49.6)	50.6 (49.8–51.4)	0.17
Single	412	51.3 (50.6–51.9)	51.5 (50.9–52.1)	0.04	48.3 (47.3–49.3)	50.8 (49.9–51.7)	0.24
Widow/divorced	96	47.2 (45.6–48.8)	45.9 (43.9–47.8)	−0.17	45.6 (43.0–48.1)	47.7 (45.2–50.4)	0.17
Education							
High school	457	51.2 (50.6–51.8)	51.2 (50.6–51.8)	—	48.1 (47.1– 49.0)	50.1 (49.2–51.0)	0.20
Secondary school	311	51.1 (50.3–51.9)	50.1 (49.4–50.9)	−0.13	48.2 (47.1–49.4)	51.2 (50.0–52.3)	0.28
Primary school	227	49.0 (48.2–49.9)	47.5 (46.4–48.5)	−0.23	49.0 (47.6–50.4)	50.7 (49.2–52.1)	0.16
Degree	143	52.7 (51.8–53.6)	52.4 (51.4–53.4)	−0.05	49.0 (47.4–50.7)	50.6 (49.2–52.1)	0.16
None	22	46.8 (42.6–50.9)	44.9 (41.2–48.5)	−0.19	47.7 (42.9–52.6)	43.1 (37.5–48.7)	−0.39
Working status							0.21
Worker	632	51.7 (51.2–52.2)	51.5 (51.0–52.0)	−0.04	49.2 (48.4–49.9)	51.2 (50.5–52.0)	0.07
Retired	222	49.2 (48.3–50.1)	47.6 (46.6–48.7)	−0.23	50.8(49.5–52.1)	51.5 (50.0–53.0)	0.20
House keeper	181	48.9 (47.8–50.0)	47.8 (46.6–49.0)	−0.15	44.9 (43.2–46.5)	47.2 (45.5–48.9)	0.26
Student	65	51.6 (50.1–53.0)	52.3 (51.0–53.6)	0.12	47.0 (44.6–49.4)	49.7 (47.6–51.8)	0.41
Unemployed	36	52.2 (50.5–53.9)	51.1 (49.5–52.7)	−0.16	43.9 (41.1–46.7)	48.7 (46.3–51.0)	
Systemic diseases							
No	933	51.3 (50.9–51.7)	51.0 (50.6–51.5)	−0.04	48.5(47.9– 49.2)	50.6 (50.0–51.3)	0.20
Yes	249	49.1 (48.1–50.0)	47.5 (46.4–48.5)	−0.21	48.1 (46.8–49.5)	49.6 (48.1–51.0)	0.13
*Unreported *	*41*						
Clinical indications							
Ectasia/thinning	721	51.7 (51.2–52.1)	51.3 (50.8–51.7)	−0.07	48.4 (47.6–49.1)	50.7 (50.0–51.4)	0.23
All other indications	502	49.5 (48.9–50.2)	48.7 (48.0–49.4)	−0.12	48.4 (47.6–49.1)	50.1 (49.0–51.1)	0.14
Previous keratoplasties							
No	1,020	50.9 (50.5–51.3)	50.3 (49.8–50.7)	−0.09	48.3 (47.7–49.0)	50.5 (49.8–51.1)	0.20
Yes	148	50.1 (48.9–51.3)	49.4 (48.1–50.6)	−0.09	48.4 (46.5–50.2)	49.5 (47.6–51.5)	0.10
* Unreported*	*55*						
Previous ocular surgery (*other than graft*)							
No	884	51.5 (51.0–51.9)	50.8 (50.4–51.3)	−0.10	48.2 (47.5–48.9)	50.5 (49.8–51.1)	0.22
Yes	284	48.8 (47.9–49.6)	48.1 (47.0–49.1)	−0.09	48.9 (47.5–50.2)	50.1 (48.7–51.5)	0.10
* Unreported*	*55*						
Other ocular diseases							
No	939	51.0 (50.6–51.5)	50.6 (50.1–51.0)	−0.07	48.0 (47.4–48.7)	50.2 (49.6–51.0)	0.20
Yes	257	50.0 (49.1–50.9)	49.1 (48.2–50.1)	−0.12	49.4 (48.1–50.7)	51.0 (49.7–52.3)	0.15
* Unreported*	*27*						
Adverse reactions/complications (*follow-up*)							
No	809	50.9 (50.4–51.4)	50.7 (50.3–51.2)	−0.02	48.7 (48.0–49.4)	50.9 (50.2–51.6)	0.21
Yes	188	51.6 (50.6–52.5)	49.9 (48.8–51.0)	−0.24	47.3 (45.8–48.7)	48.9 (47.3–50.6)	0.16
* Unreported*	*226*						
BCVA^§^ (*at baseline*)							
≤2/10	922	50.7 (50.3–51.2)	50.4 (49.6–50.5)	−0.10	48.5 (47.9–49.2)	50.6 (49.9–51.3)	0.20
3/10-4/10	188	50.8 (49.9–51.7)	51.1 (50.2–52.0)	0.04	48.6 (47.1–50.1)	49.8 (48.2–51.3)	0.11
5/10-7/10	76	51.7 (50.1–53.4)	50.4 (48.8–52.0)	−0.18	46.8 (44.0–49.6)	50.7 (48.4–52.9)	0.31
≥8/10	5	52.1 (43.3–60.9)	47.7 (37.5–57.9)	−0.62	50.9 (34.4–67.3)	55.9 (48.1–63.7)	0.38
* Unreported*	*32*						

*Negative values of ES indicate poorer SF-12 scores compared to baseline; ^§^best corrected visual acuity.

**Table 5:** Patients' expectation and satisfaction.

Questions	Yes *n* (%)	No *n* (%)	Uncertain *n* (%)
(1) Are you happy with your graft outcome?	1,016 (83.1)	144 (11.8)	63 (5.1)
(2) Does the outcome match your expectation?	864 (70.6)	293 (24.0)	66 (5.4)
(3) Would you make the same decision again?	1,100 (90.0)	65 (5.3)	57 (4.7)
(4) Was having the graft worthwhile?	1,170 (95.7)	27 (2.2)	26 (2.1)
(5) Have you had more complications than you expected?	271 (22.2)	948 (77.5)	4 (0.3)

**Table 6:** Overall patients' satisfaction.

	Males *n* (%)	Females *n* (%)	Total *n* (%)
Completely	411 (57.2)	268 (53.2)	679 (55.5)
Quite	217 (30.2)	166 (33.0)	383 (31.3)
None	68 (9.5)	58 (11.5)	126 (10.3)
Uncertain	23 (3.1)	12 (2.3)	35 (2.9)

Total	719 (100.0)	504 (100.0)	1.223 (100.0)

## Abbreviations

(HRQOL): Health-related quality of life
(SF-12): 12-item short-form health survey
(PK): Penetrating keratoplasty
(ALK): Anterior lamellar keratoplasty
(PCS-12): Physical component summary
(MCS-12): Mental component summary
(ES): Effect size.
